# Anti-CD3 Antibody Treatment Induces Hypoglycemia and Super Tolerance to Glucose Challenge in Mice through Enhancing Glucose Consumption by Activated Lymphocytes

**DOI:** 10.1155/2014/326708

**Published:** 2014-02-11

**Authors:** Chang-Qing Xia, Anna V. Chernatynskaya, Benjamin Looney, Suigui Wan, Michael J. Clare-Salzler

**Affiliations:** ^1^Department of Hematology, Xuanwu Hospital, Capital Medical University, No. 45 Changchun Street, Xicheng District, Beijing 100053, China; ^2^Department of Pathology, Immunology and Laboratory Medicine, University of Florida College of Medicine, Gainesville, FL 32610, USA

## Abstract

Anti-CD3 antibody has been employed for various immune-mediated disorders. However, whether anti-CD3 administration leads to rapid metabolic alternation has not been well investigated. In the current study, we studied how anti-CD3 treatment affected blood glucose levels in mice. We found that anti-CD3 treatment induced immediate reduction of blood glucose after administration. Furthermore, a single dose of anti-CD3 treatment corrected hyperglycemia in all nonobese diabetic mice with recently diagnosed diabetes. This glucose-lowering effect was not attributable to major T cell produced cytokines. Of interest, when tested in a normal strain of mice (C57BL/6), the serum levels of C-peptide in anti-CD3 treated animals were significantly lower than control mice. Paradoxically, anti-CD3 treated animals were highly tolerant to exogenous glucose challenge. Additionally, we found that anti-CD3 treatment significantly induced activation of T and B cells *in vitro* and *in vivo*. Further studies demonstrated that anti-CD3 treatment lowered the glucose levels in T cell culture media and increased the intracellular transportation of 2-(N-(7-nitrobenz-2-oxa-1,3-diazol-4-yl)amino)-2 deoxyglucose (2-NBDG) particularly in activated T and B cells. In addition, injection of anti-CD3 antibodies induced enhanced levels of Glut1 expression in spleen cells. This study suggests that anti-CD3 therapy-induced hypoglycemia likely results from increased glucose transportation and consumption by the activated lymphocytes.

## 1. Introduction

Anti-CD3 antibody therapy has been employed in several immune-mediated disorders, such as type 1 diabetes (T1D). Animal studies have demonstrated that anti-CD3 therapy not only prevents diabetes but also reverses new onset diabetes in nonobese diabetic (NOD) mouse model. The results of the clinical trials on T1D anti-CD3 therapy showed its effect on improving C-peptide levels. The mechanisms underlying the effectiveness of anti-CD3 therapy have been characterized including activation-induced T cell death [1], T cell anergy [2] and Foxp3+ Treg cell induction [3–5], and induction of tolerogenic antigen-presenting cells through processing dead T cells induced by anti-CD3 therapy [6]. However, whether anti-CD3 therapy induces immediate effect on blood glucose has not been well investigated.

The purpose of anti-CD3 therapy in managing allograft rejection and autoimmune diseases is to eliminate antigen-reactive T cells through eliminating T cells by complement-mediated cell killing, or activation-induced cell death. However, one of the early events occurring right after anti-CD3 injection is that almost all T cells are activated and quickly release various soluble factors leading to cytokine storm [7]. Certain cytokines released by the activated T cells may subsequently affect metabolic process of the organism including glucose metabolism. It was reported that anti-CD3 treatment induced hypothermia and hypoglycemia in normal strains of mice [8], and cytokines such as IFN-*γ* and TNF-*α* were shown to be responsible for the hypoglycemia induced by anti-CD3 treatment [8, 9]. Due to the complexity of anti-CD3 therapy, the effect of cytokines on anti-CD3-induced hypoglycemia needs to be further evaluated. Given that glucose metabolism alters in activated T cells, the alterations of glucose metabolism in anti-CD3 treatment induced activated T cells may also contribute to the hypoglycemia in anti-CD3 treated animals. Furthermore, it would be of interest to know whether anti-CD3 treatment has such immediate glucose-lowering effect in diabetic mice and whether this therapy influences the sensitivity to glucose challenge.

In the present study, we examined the immediate effect of anti-CD3 treatment on blood glucose in normal strain of mice (C57BL/6), new onset diabetic NOD mice. We confirmed the previous reports [8] by showing that anti-CD3 Ab lowered blood glucose levels around 4 hours following injection but failed to reproduce the results that anti-cytokine antibodies reversed hypoglycemia induced by anti-CD3 Ab therapy. Of interest, we found that a single dose of anti-CD3 treatment was able to correct the hyperglycemia in new onset diabetic NOD mice and this effect lasted for as long as 3 days. Intriguingly, animals receiving anti-CD3 treatment acquired super tolerance to glucose challenge but paradoxically exhibited reduced levels of serum C-peptide.

## 2. Methods and Materials

### 2.1. Experimental Animals

C57BL/6 mice (age of 6–8 weeks) and nonobese diabetic (NOD) mice and NOD-Rag^−/−^ mice were purchased from Jackson Laboratory, or Chiles River in China. All mice were maintained under specific pathogen-free conditions and used following the governmental and institutional guidelines for animal welfare.

### 2.2. Administration of Anti-CD3 Antibodies and Dynamic Observation of Blood Glucose

Anti-CD3 antibodies (clone: 145-2C11, purchased from BD Bioscience) were diluted in PBS (1 *μ*g/*μ*L). Fifty microliter of the diluted anti-CD3 solution was injected intraperitoneally to each mouse. For normal strain of mice, blood glucose was measured by Accu-check Glucometer every two hours in the first 8 hours until 48 hours after anti-CD3 injection. For diabetic NOD mice, blood glucose was measured every day for the first 3 days and every other day until day 7 after anti-CD3 treatment.

### 2.3. Intraperitoneal Glucose Tolerance Test (IPGTT)

IPGTT was performed as previously described [10]. Briefly, glucose solution was injected intraperitoneally (1 g/kg body weight); then blood glucose was measured at 10, 30, 60, and 120 minutes by Accu-check Glucometer. The baseline glucose levels of individual mice before glucose injection were also recorded.

### 2.4. Measurement of Blood C-Peptide Levels

Mouse serum C-peptide levels were measured by YK013 mouse C-peptide EIA kit following the instruction from the manufacturer (Cosmo Bio Co., Ltd., Japan).*‬*

### 2.5. Measurement of Serum IFN-*γ*, TNF-*α*, IL-1*α*, and IL-6

IFN-*γ*, TNF-*α*, IL-1*α*, and IL-6 in serum samples were measured by multiplexed cytokine assay kit following the instruction from the manufacturer (Millipore). The cytokines in the above samples were examined by Luminex 100 (Luminex corporation). The collected Luminex data were analyzed using data analysis software from Brendan Technologies Inc. (Carlsbad, CA).

### 2.6. IFN-*γ* Injection on Blood Glucose

Firstly, we injected mice with mouse IFN-*γ* (purchased from PeproTech Cherry Hill, NJ) at a dose of doubled average levels of serum IFN-*γ* (30 ng/mouse) 6 hours after-anti-CD3 treatment, and blood glucose was measured using Accu-check Glucometer at 1, 2, 4, 6, and 24 hours after IFN-*γ* injection. It was noted that there was no change in terms of blood glucose levels after IFN-*γ* treatment. Then, we tested higher dose of IFN-*γ* (200 ng/mouse) in the above mice and monitored blood glucose at 1, 2, and 4 hours after IFN-*γ* injection. Since we did not observed any change in blood glucose levels after this higher dose of IFN-*γ* injection, we discontinued monitoring blood glucose levels at 4 hours after injection.

### 2.7. Neutralizing Anti-TNF-*α* Antibody Administration on Anti-CD3 Treatment Induced Hypoglycemia

C57BL/6 mice were treated with anti-CD3 antibodies (50 *μ*g/mouse), or isotype IgG (50 *μ*g/mouse). At 2 and 24 hours after the treatment, anti-CD3 treated mice were treated with neutralizing anti-TNF-*α* antibodies (BioLegend) or isotype IgG (BioLegend) (50 *μ*g/mouse). Blood glucose levels of all mice were monitored using Accu-check Glucometer at 1, 2, 4, 6, 24, 27, 29, 31, 47, and 70 hours after anti-CD3 treatment.

### 2.8. Neutralizing Anti-IFN-*γ* Antibody Administration on Anti-CD3 Treatment Induced Hypoglycemia

C57BL/6 mice were treated with anti-CD3 antibodies (50 *μ*g/mouse) and then received intraperitoneal injection of neutralizing anti-IFN-*γ* (BioLegend) or isotype IgG (BioLegend) (50 *μ*g/mouse). Blood glucose levels of all mice were monitored at 2, 4, 6, 23, and 31 hours after treatment.

### 2.9. Neutralizing Anti-IL-6 Antibody Administration on Anti-CD3 Treatment Induced Hypoglycemia

C57BL/6 mice were treated with anti-CD3 antibodies (50 *μ*g/mouse) and then received intraperitoneal injection of neutralizing anti-IL-6 (BioLegend) or isotype IgG (BioLegend) (50 *μ*g/mouse). Blood glucose levels of all mice were monitored at 2, 4, 6, 7, 22, 24, 26, and 27 hours after treatment.

### 2.10. Test of Anti-CD3 Treatment on T Cell and B Cell Activation *In Vitro* and *In Vivo*

*In Vitro Experiment*. Freshly prepared spleen cells 1 × 10^6^/well in 24-well plate were stimulated with anti-CD3 antibodies (3 *μ*g/mL) or Isotype IgG for 24 hours. Thereafter, The cells were harvested and stained with CD4, CD8, B220, and CD69 and analyzed by flow cytometry.

*In Vivo Experiment*. C57BL/6 mice were treated with intraperitoneal injection of anti-CD3 antibodies (50 *μ*g/mouse) or Isotype IgG (50 *μ*g/mouse). Twenty-four hours later, all mice were killed, and spleen cells of each individual mouse were prepared and stained for CD4, CD8, B220, and CD69. The expression of these markers were examined by flow cytometry.

### 2.11. Examination of the Influence of Lymphocyte Activation on Glucose Uptake *In Vitro*

Spleen cells from C57BL/6 mice were freshly prepared. Spleen cells (1 × 10^6^/well/200 *μ*L) were cultured in complete RPMI media containing glucose 360 mg/dL, with addition of anti-CD3 antibodies (3 *μ*g/mL) or isotype IgG (3 *μ*g/mL) for 24 hours. The culture for each condition was quadruplicated. Glucose concentrations in the culture media were measured at 24 hours after culture using Accu-check Glucometer. In another experimental setting, spleen cells (1 × 10^6^/well/1 mL) were cultured in a 48-well plate in complete RPMI media with anti-CD3 antibodies (3 *μ*g/mL) or isotype IgG (3 *μ*g/mL) for 3 hours; thereafter different concentrations of 2-(N-(7-nitrobenz-2-oxa-1,3-diazol-4-yl)amino)-2-deoxyglucose (2-NBDG) as indicated were added to the cultures for additional 2 hours. Quadruplicated wells were set for each condition. After the incubation, the cells were washed in the plate using cold PBS for 4 times, and then the fluorescence intensity was detected by BioTec Synergy 2 plate reader (BioTek Instrument, Inc.). For anti-CD3-stimulated wells, an aliquot of the cells was stained with CD5, B220, and CD69. 2-NBDG uptake by activated (CD69+) and nonactivated (CD69−) T cells (CD5+) and B cells (B220+) were analyzed by flow cytometry.

### 2.12. Real-Time PCR for Glut1 Expression on Spleen Cells of Mice Treated with Anti-CD3 and Isotype IgG Antibodies

B6 mice were treated with intraperitoneal injection of anti-CD3 antibodies (50 *μ*g/mouse) or isotype IgG (50 *μ*g/mouse) for 6 hrs. The expression of Glut1 in spleen cells was examined by real-time PCR following the protocol from the manufacturer (Invitrogen, Life Tech). Glut1 gene expression level in each sample was normalized by the gene expression level of *β* actin. Glut1 expression in control spleens was defined as 1; the level of Glut1 in anti-CD3 treatment group relative to control was calculated accordingly.

## 3. Results

### 3.1. A Quick Correction of Hyperglycemia by Anti-CD3 Treatment in New Onset Diabetic NOD Mice

**Figure 1:**
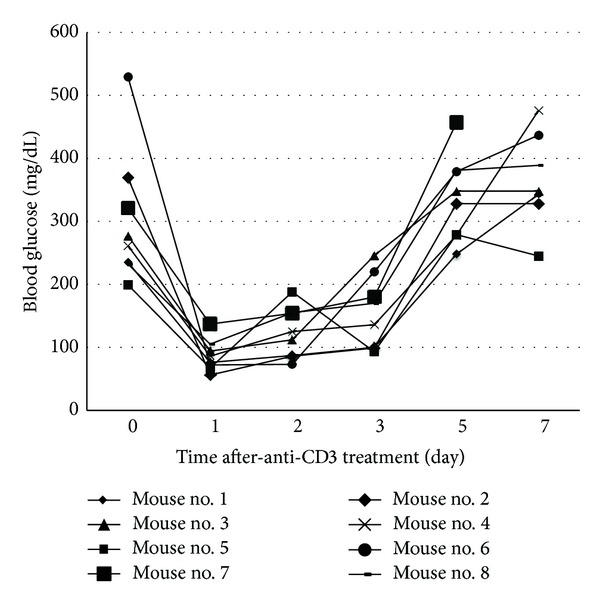
Effect of anti-CD3 treatment on blood glucose of NOD mice with new onset disease. NOD mice with blood glucose over 200 mg/dL for two consecutive days were treated with intraperitoneal injection of anti-CD3 (50 *μ*g/mouse). Thereafter, blood glucose was monitored daily for 7 days. Eight diabetic NOD mice were included in this experiment.

Anti-CD3 therapy has been showing a long-term T1D reversing effect after 5 daily injections in new onset diabetic NOD mice [11]. However, few studies have investigated how anti-CD3 antibody affects blood glucose shortly after administration. To assess the immediate effect of anti-CD3 antibody treatment in new onset diabetic NOD mice, NOD mice with blood glucose over 200 mg/dL for two consecutive days were treated with a single dose of anti-CD3 antibody. Then, blood glucose was measured daily. Surprisingly, we found that all new onset diabetic NOD mice with blood glucose levels as high as 500 mg/dL were corrected to or lower than normal levels within 24 hours (Figure 1). In some mice, this effect lasted for more than three days (Figure 1).

### 3.2. Anti-CD3 Treatment Leads to Hypoglycemia in Normal Strain of Mice

**Figure 2:**
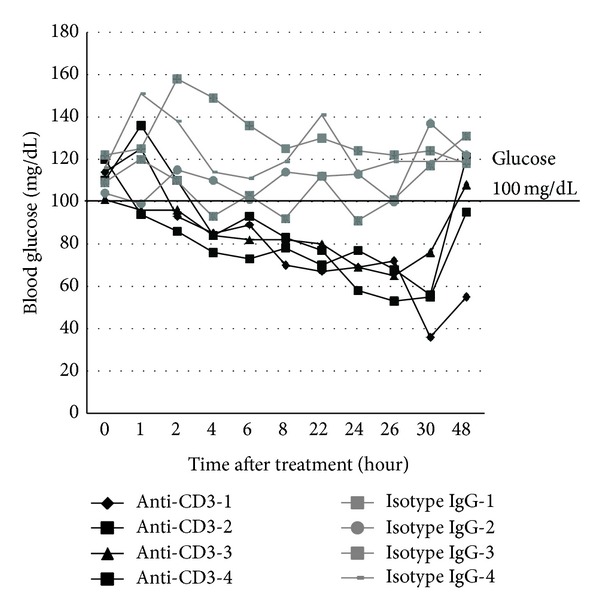
Effect of anti-CD3 treatment on blood glucose of normal strain of mice. C57BL/6 mice were treated with intraperitoneal injection of anti-CD3 antibody (50 *μ*g/mouse) or isotype IgG (50 *μ*g/mouse). Four mice were included in each group. Blood glucose levels were measured at different time points as depicted in the figure. The blood glucose changes over 48 h observation were shown.

Two decades ago, one group reported that anti-CD3 treatment led to hypoglycemia [8, 9]. There has been little follow-up research thereafter. Thus, it is worth revisiting this issue on the effect of anti-CD3 treatment on blood glucose. We treated B6 mice (H-2b) with the same dose of anti-CD3 antibodies as described above. Then, blood glucose was monitored every two hours at daytime for three days. We found that blood glucose levels in all anti-CD3 treated animals became lower than normal 4 hours after the treatment. In addition, the hypoglycemia remained for at least 48 hours and was gradually recovered by 72 hours after treatment (Figure 2). There was no difference in terms of eating and activities between the two groups. Anti-CD3 treatment did not lead to dehydration and loss of body weight (data not shown).

### 3.3. Anti-CD3 Treatment Leads to Super Tolerance to Exogenous Glucose Challenge

**Figure 3:**
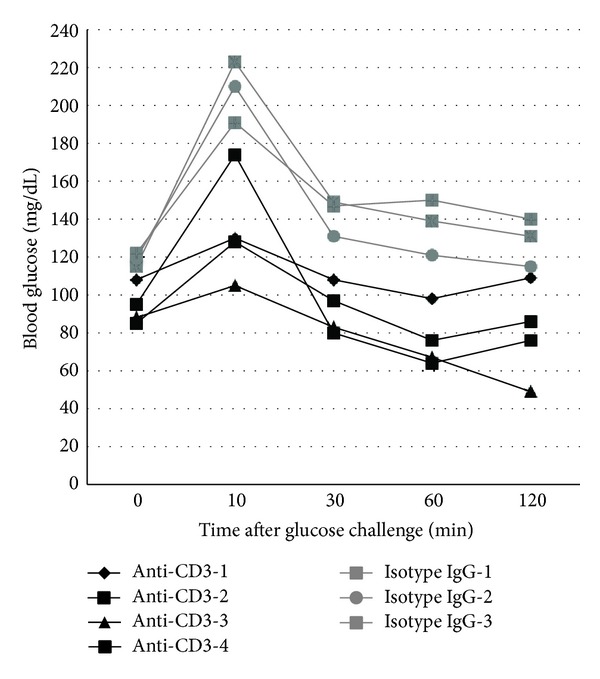
Intraperitoneal glucose tolerance test (IPGTT) in mice treated with anti-CD3 antibody or isotype IgG. C57BL/6 mice under the treatment of anti-CD3 antibody or isotype IgG as described elsewhere for 48 h received intraperitoneal injection of glucose (1 g/kg body weight). Thereafter, the blood glucose was monitored at 10, 30, 60, and 120 min. The glucose level before glucose injection served as the baseline level for each mouse. The results of all animals at different time points were depicted individually.

The above glucose-lowering effect prompted us to assess the response to glucose challenge. Animals were treated with anti-CD3 or isotype IgG. At 48 hours after the treatment, we performed intraperitoneal glucose tolerance test (IPGTT) by challenging the mice with high dose of glucose (1 g/kg body weight). As shown in Figure 3, all anti-CD3 treated mice exhibited super tolerance in contrast to control mice. In some anti-CD3 treated mice, blood glucose was only slightly elevated at 10 min after glucose injection when glucose levels in the control group reached the peak levels and quickly dropped to normal levels. These findings suggest that anti-CD3 treatment may directly or indirectly affect glucose metabolism.

### 3.4. Anti-CD3 Treatment Lowers Blood C-Peptide Levels

**Figure 4:**
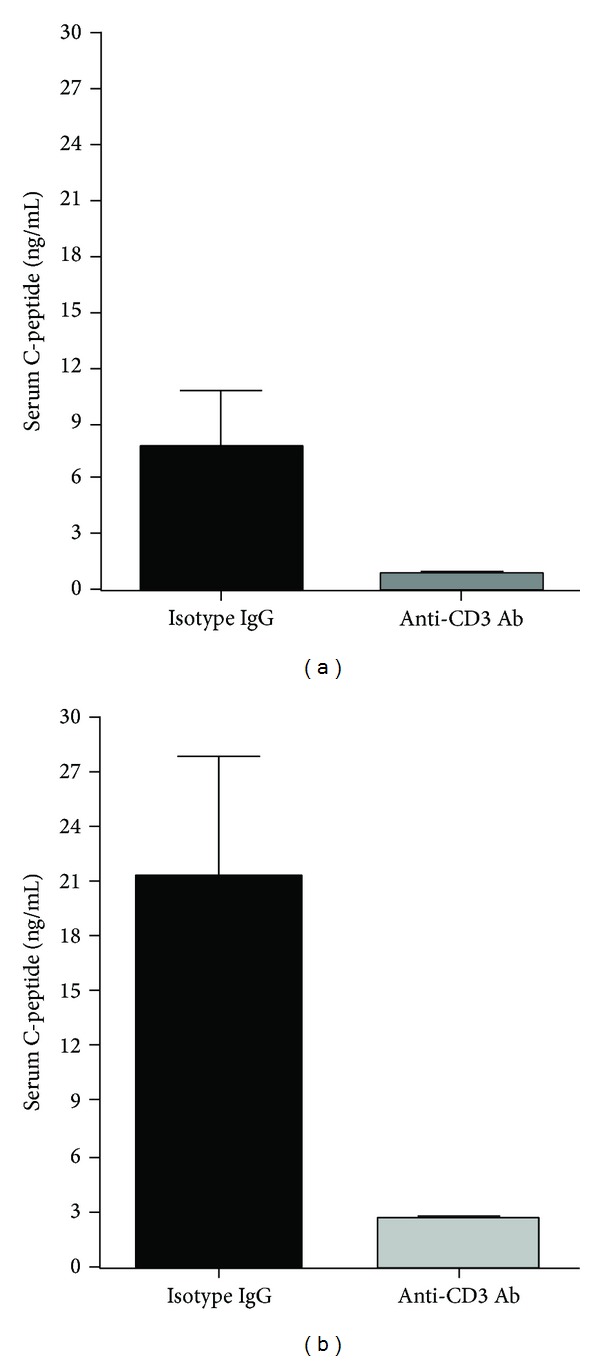
Serum C-peptide levels of the mice at 48 h after anti-CD3 or isotype IgG treatment and their levels after IPGTT. Serum samples were collected from the mice in Figure 3 before IPGTT and collected again after IPGTT. ELISA was used to measure C-peptide levels of all mice in both groups. Compared to controls, C-peptide levels of anti-CD3 treated mice were significantly lower before and after IPGTT (*P < 0.05).

The quick lowering effect on blood glucose as well as the super tolerance to glucose challenge in anti-CD3 treated mice suggests that anti-CD3 treatment might have enhanced *β* cell function in secreting insulin thereby leading to hypoglycemia. To assess whether anti-CD3 antibody treatment leads to augmentation of insulin secretion, we measured C-peptide levels 6 hours after anti-CD3 treatment. Surprisingly, we found that the levels of C-peptide in anti-CD3 treated mice were significantly lower than those of the control mice (Figure 4(a)). Paradoxical to the super glucose tolerance (Figure 3), there was no significant increase in C-peptide levels in the anti-CD3 treated mice one-day after IPGTT. In contrast, the C-peptide levels in the control mice were drastically increased one-day after IPGTT (Figure 4(b)).

### 3.5. Serum Cytokine Levels after Anti-CD3 Treatment

**Figure 5:**
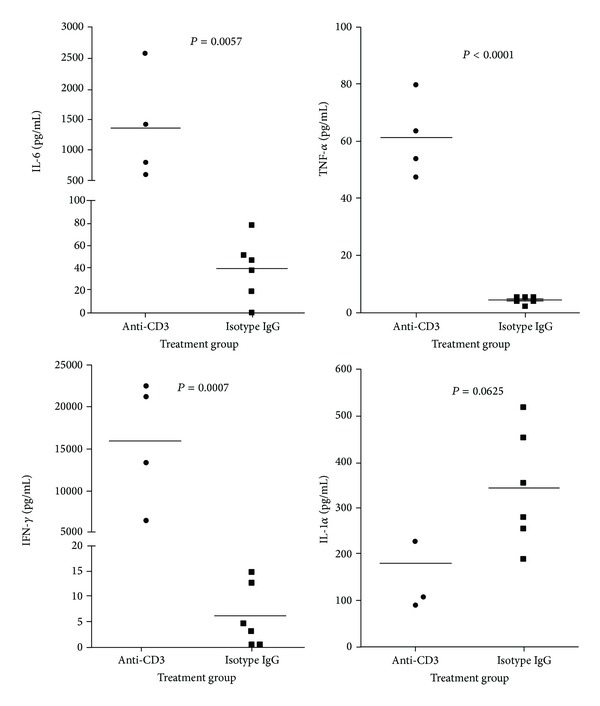
Serum cytokines in mice treated with anti-CD3 antibody or isotype IgG. C57BL/6 mice were treated with anti-CD3 (4 mice) or isotype IgG (6 mice). Six hours later, serum samples were collected for cytokine measurement. The cytokines, IFN-*γ*, TNF-*α*, IL-1, and IL-6, were examined by Luminex and the statistic analysis data were shown in the figure.

It has been known that anti-CD3 therapy can induce cytokine storm because anti-CD3 antibody can activate T cells to secrete a variety of T cell cytokines [7, 9]. Certain cytokines have been demonstrated to be able to lower blood glucose. To evaluate the cytokines induced by anti-CD3 treatment, we measured a panel of T cell cytokines in the sera of both groups at 6 and 72 hours after treatment. As shown in Figure 5, six hours after treatment, in contrast to the control animals, those receiving anti-CD3 treatment had significantly increased levels of IFN-*γ*, IL-6, and TNF-*α* particularly IFN-*γ* but there was no difference in IL-1*α* levels (Figure 5). Seventy-two hours after treatment, serum levels of all cytokines measured were low and comparable between the two groups (data not shown). The above findings suggest that the initial cytokine production by the anti-CD3 activated T cells may be responsible for the quick drop of blood glucose in anti-CD3 treated animals.

### 3.6. Major Proinflammatory T Cell Cytokines TNF-*α*, IL-6, and IFN-*γ* Are Not Responsible for Hypoglycemia Induced by Anti-CD3 Treatment

**Figure 6:**
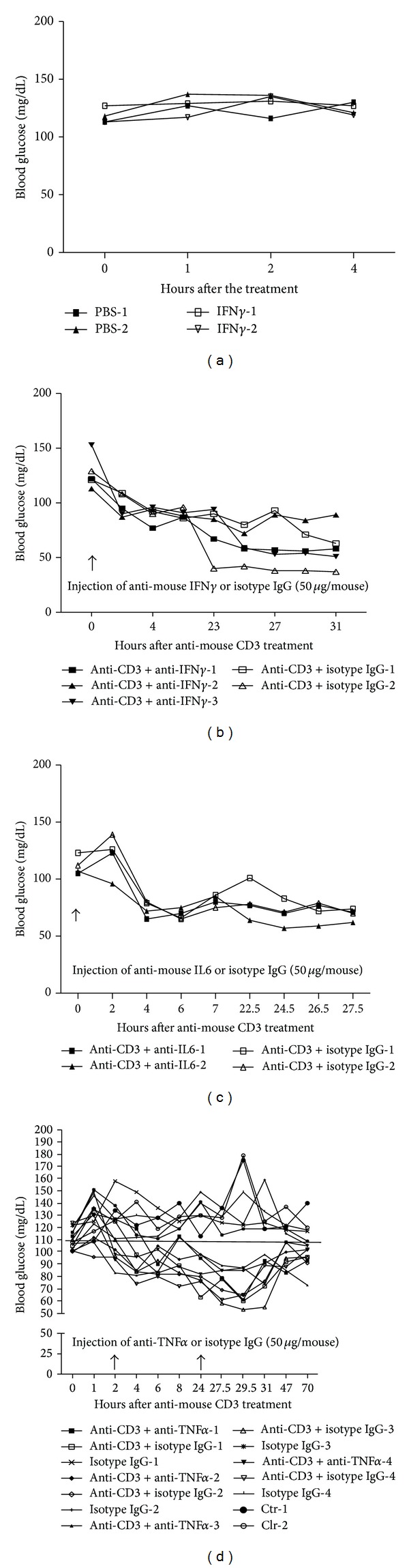
The influence of cytokines on blood glucose. (a) C57BL/6 mice were treated with intraperitoneal injection of a high dose of IFN-*γ* (200 ng/mouse) or PBS. Thereafter, the blood glucose level was monitored at different time points as shown in the figure; (b) C57BL/6 mice were treated with intraperitoneal injection of anti-CD3 antibody (50 *μ*g/mouse) plus anti-IFN-*γ* antibody (50 *μ*g/mouse), or anti-CD3 antibody (50 *μ*g/mouse) plus isotype IgG (50 *μ*g/mouse). Thereafter, the blood glucose level was monitored at different time points as shown in the figure; (c) C57BL/6 mice were treated with intraperitoneal injection of anti-CD3 antibody (50 *μ*g/mouse) plus anti-IL-6 antibody (50 *μ*g/mouse), or anti-CD3 antibody (50 *μ*g/mouse) plus isotype IgG (50 *μ*g/mouse). Thereafter, the blood glucose level was monitored at different time points as shown in the figure; (d) C57BL/6 mice were treated with intraperitoneal injection of anti-CD3 antibody (50 *μ*g/mouse for 8 mice) or isotype IgG (50 *μ*g/mouse for 4 mice). Anti-CD3 group was further divided into two subgroups with one group (4 mice) receiving anti-TNF-*α* (50 *μ*g/mouse) and the other group (4 mice) receiving isotype IgG (50 *μ*g/mouse) at 0 and 24 h time points. Additionally, two control mice without any treatment were included in this experiment. Thereafter, the blood glucose level was monitored at different time points as shown in the figure.

In these experiments, we attempted to determine the roles of cytokines induced by anti-CD3 treatment in anti-CD3-induced hypoglycemia. Based on the levels of the cytokines measured above, serum IFN-*γ* levels were drastically elevated in anti-CD3 treated animals, and IL-6 was at the second highest levels. To determine whether IFN-*γ* was responsible for the hypoglycemia in the anti-CD3 treated animals, firstly, we injected recombinant mouse IFN-*γ* at a dose of doubled average serum level of IFN-*γ* six hours after anti-CD3 treatment (shown in Figure 5(a)), we failed to observe any difference between IFN-*γ* and PBS treated groups through 24 hours' observation (data not shown). We thought that the IFN-*γ* dose used might not be enough to cause hypoglycemia; we treated those animals with a dose of IFN-*γ* as high as 6 times of serum IFN-*γ* levels at six hours after anti-CD3 treatment. Again, we did not observe any drop in blood glucose in IFN-*γ* treated animals through closely monitoring (Figure 6(a)). The above findings suggest that IFN-*γ* induced by anti-CD3 Ab therapy is unlikely to be the factor leading to hypoglycemia.

Secondly, to further eliminate the IFN-*γ*'s role in anti-CD3 treatment induced hypoglycemia, we assessed the effect of simultaneous injection of anti-IFN-*γ* neutralizing antibodies on anti-CD3-induced hypoglycemia. We found that simultaneous administering neutralizing anti-IFN-*γ* antibodies, the same as isotype IgG, failed to reverse anti-CD3-induced hypoglycemia (Figure 6(b)). Inconsistent with the previous reports [12], our findings do not support that IFN-*γ* induced by anti-CD3 treatment is responsible for anti-CD3-induced hypoglycemia.

To assess the role of IL-6 with the second highest serum levels of anti-CD3 treated mice in anti-CD3-induced hypoglycemia, we tested how anti-IL-6 neutralizing antibodies affected blood glucose levels. The results shown in Figure 6(c) indicated that IL-6 did not contribute to the hypoglycemia induced by anti-CD3 treatment.

Alegre et al. [8] reported two decades ago that TNF-*α* was responsible for anti-CD3 treatment induced hypoglycemia in normal strain of mice. However, it was noted that the TNF-*α* levels in anti-CD3 treated mice were very low even though they were higher than those of isotype IgG treated mice (Figure 5(b)). Inconsistent with the reported results [8], we found that injection of a relatively high dose of anti-TNF-*α* neutralizing antibodies (50 *μ*g/mouse) failed to reverse hypoglycemia caused by anti-CD3 administration (Figure 6(d)). Furthermore, when we increased the dose of anti-TNF-*α* to 500 *μ*g/mouse to treat anti-CD3-injected mice, no reversal of hypoglycemia was observed (see supplemental Figure 1 in Supplementery Material available online at http://dx.doi.org/10.1155/2014/326708). Our findings indicate that TNF-*α* is not responsible for anti-CD3-induced hypoglycemia.

### 3.7. Anti-CD3 Treatment Fails to Cause Hypoglycemia in Immunodeficient Rag^−/−^ Mice

**Figure 7:**
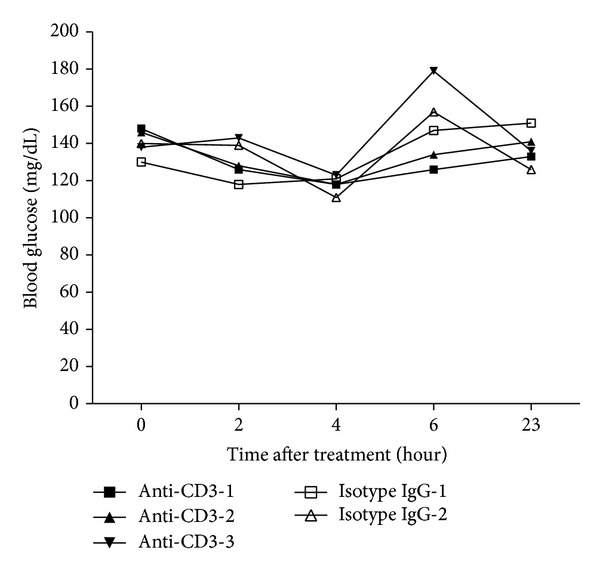
Effect of anti-CD3 treatment on blood glucose in NOD-Rag^−/−^ mice. T cell deficient NOD-Rag^−/−^ mice were treated with intraperitoneal injection of anti-CD3 antibody (50 *μ*g/mouse for 3 mice) and isotype IgG (50 *μ*g/mouse for 2 mice). Thereafter, the blood glucose level was monitored at different time points as shown in the figure.

The above findings strongly suggest that anti-CD3 treatment induced hypoglycemia is due to the alteration of biological activities of lymphocytes induced by anti-CD3 therapy. To test this, we treated NOD-Rag^−/−^ mice deficient in T and B cells with anti-CD3 antibodies or isotype IgG as described above. Blood glucose levels in both groups were monitored at 0, 4, 6, 8, and 24 hours. As shown in Figure 7, there was no significant drop in blood glucose in both groups, which indicates that the availability of lymphocytes is required, and the T cell activation by anti-CD3 antibodies is likely responsible for the development of hypoglycemia after anti-CD3 therapy.

### 3.8. Anti-CD3 Therapy Is Highly Potent in Inducing Activation of Both T and B Cells

**Figure 8:**
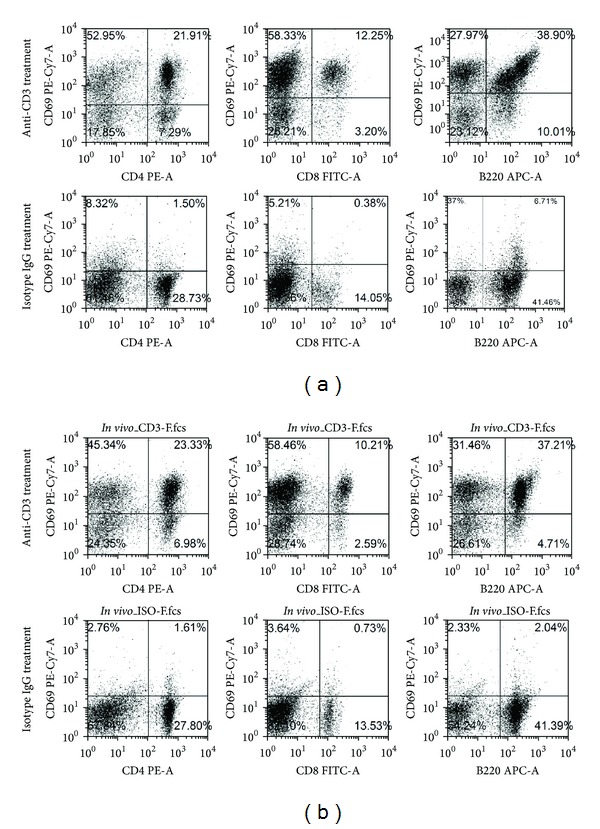
Effect of anti-CD3 antibody treatment on T and B cell activation *in vitro* and *in vivo*. (a) *In vitro experiment*. Spleen cells 1 × 10^6^/well in 24-well plate were stimulated with anti-CD3 antibodies (3 *μ*g/mL) or Isotype IgG (3 *μ*g/mL) for 24 hours. Thereafter, The cells were harvested and stained with CD4, CD8, B220, and CD69 and analyzed by flow cytometry. (b) *In vivo experiment*. C57BL/6 mice were treated with intraperitoneal injection of anti-CD3 antibodies (50 *μ*g/mouse) or Isotype IgG (50 *μ*g/mouse). Three mice were included in each group. Twenty-four hours later, all mice were killed, and spleen cells of each individual mouse were prepared and stained for CD4, CD8, B220, and CD69. The expression of these markers was examined by flow cytometry.

To assess how anti-CD3 treatment affects T and B cell activation, we stimulated mouse spleen cells *in vitro* with anti-CD3 antibodies or Isotype IgG for 24 hours. We found that most T cells including CD4 and CD8 T cells upon anti-CD3 treatment *in vitro* were activated showing upregulation of CD69 (Figure 8(a)). To our surprise, the majority of B cells were also activated (Figure 8(a)). In the cultures with addition of Isotype IgG only small percentage of T and B cells expressed CD69. To determine whether anti-CD3 therapy induces T and B cell activation *in vivo*, we injected anti-CD3 antibodies or Isotype IgG into naive mice; 24 hours later, we examined the expression of CD69 on both splenic T and B cells. The same as the *in vitro* findings, we found that T and B cells in anti-CD3 treated group significantly upregulated CD69 in contrast to Isotype IgG treated group (Figure 8(b)).

### 3.9. Anti-CD3 Treatment Enhances Glucose Transportation into Activated Lymphocytes

**Figure 9:**
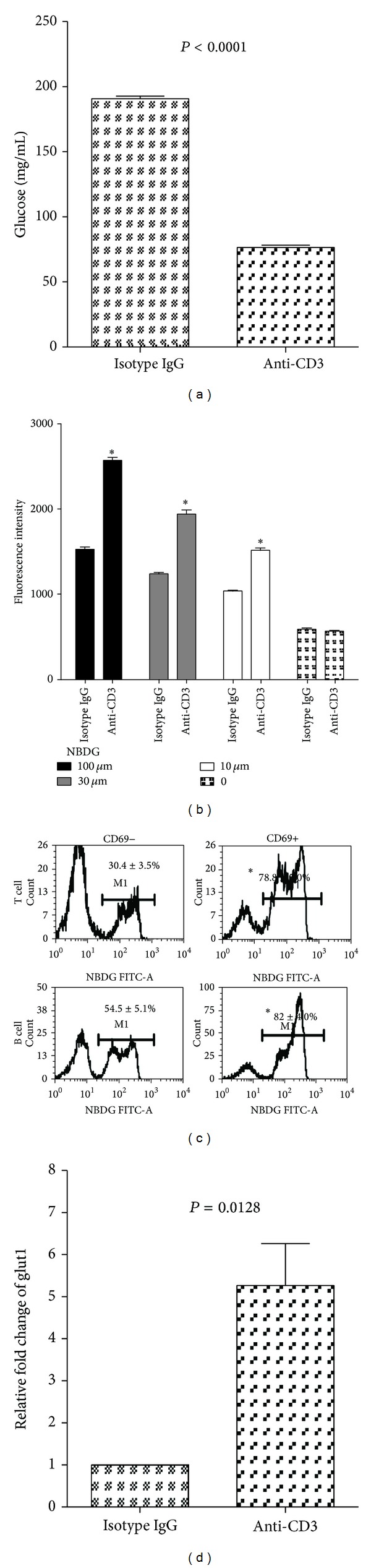
Anti-CD3 treatment enhances glucose intracellular transportation by the activated lymphocytes. (a) Freshly prepared spleen cells (1 × 10^7^/well) were cultured in a 24-well plate in 1 mL medium containing 10% fetal bovine serum with glucose concentration of 360 mg/dL. Anti-CD3 antibody (3 *μ*g/mL) or isotype IgG (3 *μ*g/mL) was added to the cultures. The culture wells were quadruplicated and incubated for 24 h. Glucose concentrations were measured before and 24 h after incubation. The results were reproduced in additional two independent experiments. (b) Freshly prepared spleen cells were stimulated with anti-CD3 antibody (3 *μ*g/mL) or isotype IgG (3 *μ*g/mL) for 3 hours; thereafter, different concentrations of 2-NBDG as indicated were added to the cultures for additional 2 hours. The fluorescent intensity of the cells was examined by BioTec plate reader using a laser filter with 480 nm excitation and 528 nm emission. The values of between anti-CD3 and isotype IgG were compared for each concentration of 2-NBDG (*P < 0.01). The similar results were reproduced in additional 3 independently experiments. (c) Spleen cells were stimulated with anti-CD3 antibodies as mentioned above for 3 hours, and then 100 *μ*M of 2-NBDG was added to the cultures for additional 2 hours. Thereafter, the cells were harvested and stained with CD5-PerCp, B220-PE, and CD69-PE-Cy7. 2-NBDG+ cells in CD69− and CD69+ T cells and B cells were analyzed through gating CD5+ and B220+ cells, respectively. The percentages of 2-NBDG+CD69− and 2-NBDG+CD69+ T or B cells were compared, respectively (*P < 0.01). The data shown were obtained from a representative of three experiments. (d) B6 mice were treated with anti-CD3 (50 *μ*g/mouse) or isotype IgG (50 *μ*g/mouse). Three mice were included in each group. Six hours later, the spleen cells were prepared and Glut1 expression was examined by real-time PCR. The values were normalized by housekeeping gene *β* actin, and the expression of Glut1 for isotype IgG treated mice was defined as 1. Data shown were obtained from 3 mice in each group.

From the results shown above, it is likely that anti-CD3 treatment induced hypoglycemia is due to the glucose redistribution, for example, increased intracellular transportation thereby augmenting glucose metabolism. Evidence shows that T cell activation by anti-CD3 antibodies may alter T cell glucose metabolism by increasing glucose transportation into the cells and enhancing the levels of glycolysis [13]. To address this issue, we utilized *in vitro* systems to assess how anti-CD3 stimulation affected glucose uptake by spleen cells. Data in Figure 9(a) clearly showed that anti-CD3 stimulation led to significant reduction of glucose concentrations in the culture media in contrast to isotype IgG treated cultures. In line with this finding, spleen cells activated by anti-CD3 antibodies significantly increased the uptake of 2-NBDG (Figure 9(b)). To further assess whether the activated T and B cells acquire the enhanced capability of taking up glucose, we compared the ability of CD69+ and CD69− T cells or B cells to take up fluorescent 2-NBDG. As shown in Figure 9(c), in anti-CD3 treated cultures, CD69+ T cells and CD69+ B cells (right panel) took up significantly more 2-NBDG than their counterparts, CD69− T cells and CD69− B cells (left panel), respectively. To determine whether activation of lymphocytes induced by *in vivo* administration of anti-CD3 antibodies upregulates glucose transporter gene expression, we examined glut1 expression in spleen cells from anti-CD3 or isotype IgG treated mice using real-time PCR. We found that Glut1 expression in spleen cells of anti-CD3 treated mice was significantly upregulated compared to isotype IgG treated mice (Figure 9(d)).

## 4. Discussion

In the present study, we have demonstrated the immediate effect of anti-CD3 treatment on blood glucose, and for the first time it is shown that anti-CD3 treatment quickly lowers the blood glucose levels in diabetic NOD mice. Although anti-CD3 therapy has been employed in autoimmune diabetes for many years including recent clinical trials [14–16], the effect shortly after administering anti-CD3 antibody has not drawn much attention. The standard protocol of anti-CD3 therapy in NOD mice is daily injection of anti-CD3 antibodies for 5 days. Most studies were focused on observation of long-term effect of anti-CD3 therapy in preventing and reversing T1D. In fact, anti-CD3 treatment indeed influenced blood glucose immediately after administration in both nondiabetic and diabetic mice. Kinetic observation of blood glucose levels demonstrated that a single dose of anti-CD3 treatment was able to correct hyperglycemia for as long as 3 days in new onset diabetic NOD mice. It has been reported that anti-CD3 treatment can induce cytokine storm because of the activation of T cells by anti-CD3 antibodies [7]. It was also reported that certain cytokines such as IFN-*γ*, IL-6, or TNF-*α* was responsible for the induced hypoglycemia in normal strains of mice [8, 12]. However, the high efficacy of anti-CD3 treatment in correcting hyperglycemia appeared unlikely to be attributed to cytokine storm because neutralization of the candidate cytokines was unable to reverse hypoglycemia induced by anti-CD3 therapy (Figure 6 and supplemental Figure 1). Moreover, it is difficult to explain the hypoglycemia shortly after anti-CD3 treatment (3-4 hours) before massive cytokine production, which usually takes longer time through gene transcription and protein synthesis and cytokine secretion.

It is possible that for unknown reasons that anti-CD3 treatment leads to enhanced insulin production thereby causing hypoglycemia. To assess this possibility, we measured serum C-peptide levels of the mice in anti-CD3 treated and isotype IgG treated groups 6 hours after treatment. Unexpectedly, we observed that in anti-CD3 treatment group there were significantly lower levels of C-peptide than in controls. Of more interest, following exogenous glucose challenge, the C-peptide levels in control animals could be further elevated whereas the levels in anti-CD3 treated animals remained low. These unexpected changes of hypoglycemia and reduced insulin secretion by anti-CD3 treatment suggest that anti-CD3 therapy lowers blood glucose first, which leads to the reduction of insulin secretion as a consequence. The IPGTT results further support that anti-CD3 treatment indeed enhances glucose metabolism because most anti-CD3 treated mice did not show a high blood glucose peak during glucose tolerance test. Given that the serum T cell cytokine levels are elevated after anti-CD3 therapy, it is possible that anti-CD3 antibodies activate T cells *in vivo* resulting in increased consumption or intracellular transportation of glucose. Previous reports have demonstrated that there were elevated levels of glycolysis in activated T cells [17–19]. It is reported recently that glucose transportation is significantly increased upon T cell activation [13], which is consistent with our *in vitro* experimental data in the current study showing that spleen cells activated by anti-CD3 antibodies significantly reduce the glucose levels in the culture media and promote the glucose uptake by activated T and B cells. Consistent with the previously reported results that T cells upregulate Glut1 upon activation [20, 21], we observed that Glut1 expression was significantly upregulated in spleen cells of anti-CD3 treated mice compared to controls. It has been demonstrated that the upregulation of Glut1 is maintained for over 96 hours and then returned to baseline [21]. In line with this finding, we found that the correction of hyperglycemia in diabetic NOD mice and hypoglycemia in normal strain of mice caused by anti-CD3 antibody treatment is transient. In addition, we found that anti-CD3 treatment led to activation of most B cells in both *in vivo* and *in vitro* experiments. The activation of B cells is probably induced by the signals from CD40L expressed on activated T cells during anti-CD3 therapy. As shown in Figure 9, activated B cells, the same as activated T cells, have increased uptake of 2-NBDG. We postulate that B cell activation might be the predominant factor that induces hypoglycemia after 24 h, because T cells are largely depleted at the later time after anti-CD3 treatment (data not shown).

Taken together, anti-CD3 treatment quickly reverses new onset diabetes in NOD mice or leads to hypoglycemia in normal strain of mice probably through enhancing glucose consumption by increasing glucose intracellular transportation in activated T and B cells.

## Supplementary Material

Supplemental figure 1: High dose of anti-TNFa failed to reverse anti-CD3 treatment induced hypoglycemia. B6 mice were treated with intraperitoneal injection of isotype IgG (50 ug), anti-CD3 (50 ug), anti-CD3 (50 ug) plus anti-TNFa (500 ug), respectively. Blood glucose was measured every two hours for 8hrs.
