# Supplementary material for: Anti-CD3 Antibody Treatment Induces Hypoglycemia and Super Tolerance to Glucose Challenge in Mice through Enhancing Glucose Consumption by Activated Lymphocytes
Source: J Immunol Res. 2014 Feb 11;2014:326708. doi: 10.1155/2014/326708 (PMC3987876; doi:10.1155/2014/326708)
Supplement: Supplementary file 1 — Supplemental figure 1: High dose of anti-TNFa failed to reverse anti-CD3 treatment induced hypoglycemia. B6 mice were treated with intraperitoneal injection of isotype IgG (50 ug), anti-CD3 (50 ug), anti-CD3 (50 ug) plus anti-TNFa (500 ug), respectively. Blood glucose was measured every two hours for 8hrs. [file 326708.f1.pdf]

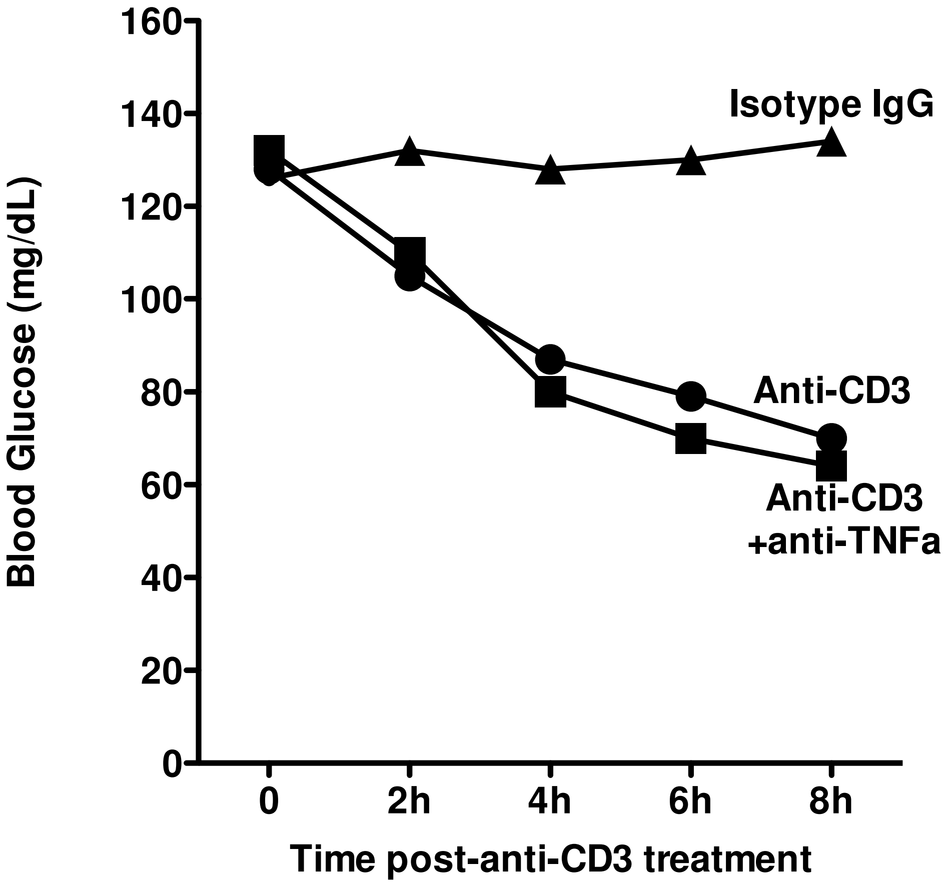

Supplemental Figure 1. High dose of anti-TNFa failed to reverse anti-CD3 treatment induced hypoglycemia. B6 mice were treated with intraperitoneal injection of isotype IgG (50 ug), anti-CD3 (50 ug), anti-CD3 (50 ug) plus anti-TNFa (500 ug), respectively. Blood glucose was measured every two hours for 8hrs.
